# Health-Related Internet Use Among Men With Prostate Cancer in Canada: Cancer Registry Survey Study

**DOI:** 10.2196/14241

**Published:** 2019-11-19

**Authors:** Jacqueline L Bender, Deb Feldman-Stewart, Christine Tong, Karen Lee, Michael Brundage, Howard Pai, John Robinson, Tony Panzarella

**Affiliations:** 1 ELLICSR Cancer Rehabilitation and Survivorship Program Department of Supportive Care Princess Margaret Cancer Centre Toronto, ON Canada; 2 Dalla Lana School of Public Health University of Toronto Toronto, ON Canada; 3 Institute of Health Policy, Management, and Evaluation University of Toronto Toronto, ON Canada; 4 Department of Oncology Queen's University Kingston, ON Canada; 5 Division of Cancer Care and Epidemiology Queen's Cancer Research Institute Kingston, ON Canada; 6 Division of Radiation Oncology BC Cancer Victoria, BC Canada; 7 Department of Surgery University of British Columbia Vancouver, BC Canada; 8 Department of Psychosocial and Rehabilitation Oncology Tom Baker Cancer Centre Calgary, AB Canada; 9 Department of Oncology University of Calgary Calgary, AB Canada

**Keywords:** prostate cancer, internet, health decision making, digital divide

## Abstract

**Background:**

After a prostate cancer diagnosis, men want information about their disease and treatment options. The internet offers a convenient means to deliver health information to patients with prostate cancer. However, there are concerns about the use of the internet among this largely senior population.

**Objective:**

This study aimed to determine the patterns and factors associated with the use of the internet as a source of health information among Canadian men with prostate cancer and the features and information required in a website.

**Methods:**

Population surveys were conducted in four Canadian provinces (British Columbia, Alberta, Saskatchewan, and Ontario) in 2014-2015. Data analyses included descriptive, bivariable, and multivariable analyses. The Pearson Chi-square and univariable regression were used to examine associations between independent variables and health-related internet use. Correlates of health-related internet use were analyzed using multivariable logistic regression.

**Results:**

A total of 1362 patients responded across the four provinces. The mean age of respondents was 69 years (SD 8.2). In addition, 82% (n=1071) were internet users and 71% (n=910) used the internet daily. Further, 65% (n=784) used the internet as a source of prostate cancer information, and 40% (n=521) were confident about using information obtained from the internet to make health decisions. Men who used the internet to obtain prostate cancer information were more likely to be active information seekers (odds ratio [OR]: 4.5, 95% CI 2.6-7.8), be confident using information from the internet to make health decisions (OR: 3.6, 95% CI 2.3-5.7), have broadband internet access (OR: 1.8, 95% CI 1.2-2.7), and have more unmet supportive care needs (OR: 1.05, 95% CI 1.0-1.1). Top features wanted in a website, reported by more than 50% of respondents, were a library of resources (n=893, 65.6%), tools to support treatment decision making (n=815, 59.8%), and tools to help navigate the prostate cancer journey (n=698, 51.2%). Top three topics of information wanted in such a website were treatment options (n=916, 67.3%), disease progression (n=904, 66.4%), and management of side effects (n=858, 63%).

**Conclusions:**

Over two-thirds of Canadian patients with prostate cancer surveyed use the internet as a source of health information about prostate cancer, but over half did not feel confident using information from the internet to make health decisions. Being an active information seeker, having confidence in using information from the internet to make health decisions, having broadband internet, and having more unmet supportive care needs were significantly associated with health-related internet use. Future work should examine electronic health literacy interventions as a means to boost men’s confidence in using information from the internet and design websites that include information and features that help men navigate the prostate cancer journey and support treatment decision making and management of side effects.

## Introduction

An estimated 1.3 million men worldwide were diagnosed with prostate cancer in 2018 [1] and 70% were from developed countries [2]. In Canada, one in seven men will be diagnosed with prostate cancer during their lifetime [3]. The risk of prostate cancer increases with age, such that 40% of all prostate cancer cases occur in men aged 60-69 years [3]. With the aging baby boom population, the number of Canadian men diagnosed with prostate cancer per year is expected to reach 42,000 by 2030 [3].

After a prostate cancer diagnosis, men want information about their disease and treatment options [4]. Information can enhance understanding, correct misconceptions about treatment, assist in coping with illness, engender feelings of control, and reduce anxiety by enabling patients to prepare for and predict aversive events [5,6]. Previous research has shown that the information needs of patients with prostate cancer are similar across time and different developed countries [4]. However, the specific amount and details of information needed varies, as do the reasons for wanting information [7].

The internet offers a convenient and cost-efficient way to provide personally tailored health information and services [8,9] and is expected to help reduce social inequalities by providing individuals access to information that might otherwise be inaccessible [10]. However, there is concern that the internet may be perpetuating disparities in access to health services by keeping certain segments of the population, such as seniors, on the sidelines [11]. According to the 2016 General Social Survey by Statistics Canada, a digital divide based on age still exists in Canada, with internet use being the lowest among those aged ≥75 years [12]. At the same time, Canadians aged 65-74 are the fastest-growing segment of internet users, with 81% using the internet in 2016 compared to 65% in 2013 [12].

Previously, we reported that for men with prostate cancer in Canada, the most frequently preferred sources of information about prostate cancer and its treatment are a urologist (96%), followed by a family doctor (90%), printed information (85%), other cancer patients (69%), and the internet (68%) [13]. These findings reflect a five-fold increase in the prevalence of health-related internet use among men with prostate in Canada compared to 10 years ago when only 12% of Canadian patients with prostate cancer used the internet as a health resource [14]. Moreover, recent data from the US Health Information National Trends survey (HINTs) shows an increasing trend toward using the internet as the first source of health information compared to family/friends/coworkers, health care professionals, and traditional media [10].

Although other studies have examined online health information–seeking behavior in the general population [10,11] and cancer survivors specifically [15], there are no recent studies on the health-related internet use of patients with prostate cancer. Current population-based data describing patterns of health-related internet use among patients with prostate cancer, as well as their confidence in using that information for making health decisions, could inform patient education and health service efforts. At the same time, understanding the factors associated with health-related internet use would be useful in designing strategies for reducing the digital divide [16,17]. In this paper, we report on the patterns and factors associated with the use of the internet as a source of health information among Canadian men with prostate cancer and the information and features wanted in a website.

## Methods

### Study Design

We conducted a cross-sectional survey using a modified Dillman [18] survey methodology, following the STROBE (Strengthening the Reporting of Observational Studies in Epidemiology) reporting standards for cross-sectional surveys [19]. Survey packages included an addressed, stamped, return envelope, which was to be returned after the survey was completed. After 4 weeks, a second survey package was sent to nonrespondents.

### Setting and Participants

We surveyed men diagnosed with prostate cancer in four Canadian provinces—British Columbia, Alberta, Saskatchewan, and Ontario—in 2014-2015 using their respective provincial cancer registries. We sought to obtain responses from 10% of the provincial patients and expected a 30% response rate. Thus, to achieve responses from 10% of the target population in each province, each registry invited a random selection of 55%-60% of men diagnosed with prostate cancer in the last 6 months of 2012 to participate in the study. We selected 2012, as it was the latest year for which the registries had complete data. The only inclusion criteria were that the patient had a prostate cancer diagnosis and lived in that province that year.

Three registries (British Columbia, Alberta, and Saskatchewan) used an “opt-out” recruitment strategy, whereby the registry provided a cover letter introducing the study in the survey package, making clear that the recipient could choose whether to complete the survey. The fourth registry (Ontario) used an “opt-in” recruitment strategy, providing a letter introducing the study and required the recipient to phone the registry to express their interest in participating. The names of interested patients from the opt-out province were then forwarded to the Ontario Lead at the study central office from where the survey was mailed out.

Each survey had a unique study identification code and did not include any identifying information. For the opt-out provinces, the provincial registries identified nonresponders and sent out the second survey 4 weeks later. For the opt-in province, the Ontario lead identified nonresponders and sent out the second survey 4 weeks later. All completed surveys were eventually mailed to the Ontario Lead at the study central office to be entered in an electronic database for analysis. Ethical approval was attained from each province’s respective university and cancer agency and from the Ontario lead’s university.

### Questionnaire

The questionnaire contained five sections with 40 questions. It was developed by a team of researchers and health care professionals and piloted with five patients prior to implementation [13]. This paper reports on questions pertaining to patterns of internet access and use (n=7), confidence using the internet as a source of health information (n=1), factors associated with health-related internet use including participant demographics (n=8), and what men with prostate cancer want in a website (n=2). All responses were self-reported.

Questions pertaining to internet access and use included use of the internet (yes/no), type of internet access (dial-up, broadband, cellular network, wireless network, or not sure), devices used to access the internet (desktop/laptop computer, tablet, cellphone/smartphone, or other), internet access from home (yes/no), frequency of internet access (at least once a day, at least once a week, at least once a month, or less than once a month), and websites used for information on prostate cancer or its treatments (open response option).

Health-related internet use was measured by asking respondents to indicate “how easy or difficult it was to obtain information about prostate cancer and/or its treatments from each of the sources below” of which, “internet (other than personal email or online support groups)” was an option. Response categories included very easy, easy, difficult, very difficult (which were all coded as “yes”), and did not try to use this source (which was coded as “no”).

Confidence in using the internet as a health resource was measured with one item from the eHealth Literacy Scale (eHEALS). The eHEALS is a validated measure of electronic health (eHealth) literacy, which is intended to capture people’s perceived skills at using information technology for health [20]. Specifically, we adapted the final question in the eHEALS scale for prostate cancer: “I feel confident in using information from the Internet to help make health decisions [related to my prostate cancer].*”* We used the same response options as the eHEALS scale: a 5-point Likert scale (1=strongly disagree, 5=strongly agree) with the addition of, “Not applicable, I do not use information from the Internet to help make decisions related to my prostate cancer.”

Lastly, preferences for using the internet as a health resource were investigated by asking participants to select information and features wanted in a website, from a list.

### Variables

We examined associations between several variables and health-related information use. *Sociodemographic variables* included age (43-65, 66-75, ≥76 years), education completed (primary or secondary school/college or university), income (≤CAD 40,000, CAD 40,001-80,000, ≥CAD 80,001), area of residence (urban or suburban/rural, town or country), and broadband internet use (yes/no). *Internet experience* variables included frequency of internet use (every day/not every day), number of devices used to access the internet (one device/more than one device), and confidence in using information from the internet to inform health decisions (as described above). *Clinical variables* included treatments (active/active surveillance or watchful waiting). *Information seeking and decision making* variables included an information-seeking role (I did all the looking, I did some of the looking along with someone else/someone else did most or all of the looking, or I did want any information), and role in the decision making process (I made the decision by myself or with my doctor, family/my doctor or a family member made the decision). *Health status variables* included overall health (very good or good/poor or very poor) and number of unmet supportive care needs, as measured by the validated 34-item Supportive Care Needs Survey and the 8-item prostate cancer–specific module [21]. Responses were no need or not applicable, yes and the need is met (met need), and yes but the need is not met (unmet need). Unmet need items were summed to create a total unmet need score.

### Data Analysis

#### Statistical Analysis

First, we carried out a descriptive analysis. Sample means and SDs were calculated for discrete/continuous variables and proportions for nominal variables. Chi-square tests and univariable logistic regression analyses were performed to test for associations between health-related internet use and variables hypothesized to be associated with such use. We assessed the construct validity of the eHealth confidence variable by examining correlations with other measures (eg, known components of eHealth literacy) in theoretically predictable ways using the Pearson Chi-square test [22]. Lastly, a multivariable logistic regression model was constructed to assess the relative importance of variables that were significantly associated (*P*<.05) with health-related internet use. Variables included in the final model were minimized through an iterative process of adding and removing variables until the model was considered to have a good fit [23]. Fit was determined based on significance values, and noting the presence or absence of interactions between variables [23].

#### Content Analysis of Open-Ended Responses

Responses to the open-ended question, “Are there any specific Internet site(s) that you like to go to for information related to prostate cancer or its treatments? If Yes, please specify,” came in the form of bulleted phrases with examples. Hence, we used content analysis to code these data into explicit categories, which we then described using statistics [24]. One coder (KL) independently reviewed and itemized all unique responses (some participants provided more than one response). A codebook was developed and a coding strategy was applied to code each unique response and organize the responses into explicit categories (eg, national cancer agencies, hospital affiliated websites, commercial websites, and online patient communities). The coding strategy was refined following discussion with the lead author (JLB). A frequency count was performed to quantify the number and proportion of responses in each category.

## Results

### Sample Characteristics

A total of 1362 patients returned partially or fully completed surveys across provinces and 46 returned blank surveys. Blank surveys and missing responses from partially completed surveys were excluded from the analysis. The survey response rate for the opt-out provinces was 46%-55% and for the opt-in province, it was 13%. Table 1 shows respondents’ sociodemographic, treatment, and general health characteristics. The age range of respondents was 43-95 years. Most were on follow-up after treatment (63%) and in good or very good health (93.6%).

**Table 1:** Respondent characteristics.

Characteristic		Count
Age (n=1320), mean (SD)		69.5 (8.2)
**Relationship status (n=1313), n (%)**		
	With partner	1134 (86)
	Without partner	179 (14)
**Sexual orientation (n=1362), n (%)**		
	Heterosexual	1201 (88.2)
	Gay	17 (1.2)
	Bisexual	8 (0.6)
**Education (highest level completed) (n=1312), n (%)**		
	Primary or secondary school	439 (33)
	College, technical school, or university	873 (67)
**Residence (n=1320), n (%)**		
	Urban or suburban	833 (63)
	Rural, town, or country	487 (37)
**Household income (n=1215), n (%)**		
	<CAD 40,000	347 (29)
	CAD 40,001-80,000	454 (37)
	>CAD 80,000	414 (34)
**Language preference for health information (n=1302), n (%)**		
	English	1257 (96.5)
	English and other language	18 (1.5)
	Other language	27 (2.3)
**Treatments received (n=1362), n (%)**		
	Surgery	549 (40.3)
	External beam radiation therapy	428 (31.4)
	Hormone therapy or androgen deprivation therapy	343 (25.2)
	Brachytherapy	240 (17.6)
	Active surveillance	210 (15.4)
	Watchful waiting	150 (11)
	Chemotherapy	27 (2)
	Complementary and alternative therapy	31 (2.3)
	High-frequency ultrasound therapy	19 (1.4)
	Cryotherapy	12 (0.9)
	Immune therapy	2 (0.1)
	Other	89 (6.3)
	I don’t know	19 (1.4)
**Stage of cancer journey (n=1139), n (%)**		
	Follow-up monitoring after treatment	719 (63)
	On active surveillance or watchful waiting	292 (26)
	Recently finished treatment, but have not had any follow-up visits	47 (4)
	Currently getting treatment for recurrent cancer	36 (3)
	Finished treatment for recurrent cancer (less than 3 months)	24 (2)
	Receiving treatment for metastatic disease	23 (2)
	Other	242 (17.2)
**General health (n=1316), n (%)**		
	Very good	506 (38.4)
	Good	727 (55.2)
	Poor	77 (5.9)
	Very poor	6 (0.5)

### Patterns of Internet Use

Table 2 shows the patterns of internet access and use. A total of 82% of respondents were internet users. The majority of respondents (70.7%) used the internet at least once a day, had home internet access (84.3%), and accessed the internet from a desktop or laptop computer (75.7%) through broadband or wireless (47%) internet connection.

### Health-Related Internet Use and Confidence

As shown in Table 2, 65% (n=784) reported that they used the internet as a source of information about prostate cancer. With respect to confidence in using information from the internet to help make decisions related to prostate cancer, 40.2% (n=521) of the respondents agreed or strongly agreed with the statement, “I feel confident in using information from the Internet to help make health decisions related to my prostate cancer,” 33.5% (n=386) were undecided or disagreed, and about one-quarter (26.3%; n=341) reported that they did not use information from the internet to make decisions related to their prostate cancer. Findings from hypothesis testing provide support of the construct validity of the single item eHealth confidence variable. eHealth confidence was positively correlated with the frequency of internet use (r=0.1, *P*=.003) and negatively correlated with “not being comfortable using a computer or mobile device” (r=–0.15, *P*<.001). In addition, eHealth confidence was negatively correlated with known components of eHealth literacy when framed as barriers. These include “not knowing how to judge the quality of the information or what information to trust” (r=–0.2, *P*<.001), “not knowing how what information applied to me” (r=–0.2, *P*<.001), “not knowing how or where to search for information” (r=–0.2, *P*<.001), and “having difficulty finding information that I could understand” (r=–0.2, *P*<.001).

**Table 2:** Patterns of internet use (n=1362).

Characteristic		Count, n (%)
Internet use (n=1310)		1071 (81.8)
**Frequency of internet use** **(n=1320)**		
	At least once a day	910 (68.9)
	At least once a week (but not every day)	143 (10.8)
	At least once a month (but not every week)	25 (1.9)
	Less than once a month	15 (1.1)
**Type of internet access** **(n=1362)**		
	A regular dial-up telephone line	61 (4.5)
	Broadband such as DSL^a^ or cable	640 (47.0)
	A cellular network (eg, cell or smartphone)	260 (19.1)
	A wireless network (Wi-Fi)	639 (46.9)
	Not sure	20 (1.5)
**Devices used to access internet** **(n=1362)**		
	Computer (desktop/laptop)	1031 (75.7)
	Tablet	465 (34.1)
	Cell phone or smart phone	411 (30.2)
Home internet access (n=1286)		1084 (84.3)
Used the internet as a source of prostate cancer information (n=1203)		784 (65.1)
Confident using information from the internet to help make decisions about prostate cancer (n=1296)		521 (40.2)

^a^DSL: digital subscriber line (the way a computer connects to the internet at high speeds using telephone lines).

### Factors Correlated With Health-Related Internet Use

As shown in Table 3, factors that correlated significantly (*P*<.05) with health-related internet use were younger age, higher education, higher income, urban residence, broadband internet access, frequency of internet use, accessing the internet from multiple devices, confidence in using information from the internet to help make health decisions related to prostate cancer, active information seeking, active decision making role, and greater number of unmet supportive care needs.

**Table 3:** Factors associated with health-related internet use (total number of health-related internet users=784/1203 valid responses).

Variables (with number of valid responses for both variables) and value		Health-related internet use		*P* value
		Yes	No	
**Age (years; n=1173), n (%)**				<.001
	43-65	316 (26.9)	79 (6.7)	
	66-75	340 (29.0)	196 (16.7)	
	≥76	108 (9.2)	134 (11.4)	
**Education (n=1169), n (%)**				<.001
	Primary/secondary	176 (15.1)	180 (15.4)	
	College or university	587 (50.2)	226 (19.3)	
**Income (n=1091), n (%)**				<.001
	<CAD 40,000	136 (12.5)	137 (12.6)	
	CAD 40,001-CAD 80,000	274 (25.1)	145 (13.3)	
	≥CAD 80,001	307 (28.1)	92 (8.4)	
**Area of residence (n=1174), n (%)**				.005
	Rural, town, or country	252 (21.5)	167 (14.2)	
	Urban or suburban	516 (44.0)	239 (20.4)	
**Broadband use (n=1203), n (%)**				<.001
	Yes	478 (39.7)	132 (11.0)	
	No	306 (25.4)	287 (23.9)	
**Internet use frequency (n=1015), n (%)**				<.001
	Every day	662 (65.2)	197 (19.4)	
	Not every day	91 (9.0)	65 (6.4)	
**Number of devices (n=962), n (%)**				<.001
	Computer only	284 (29.5)	137 (14.2)	
	Computer+mobile device	435 (45.2)	106 (11.0)	
**Electronic health confidence (n=903), n (%)**				<.001
	Confident	441 (48.8)	58 (6.4)	
	Not confident	284 (31.5)	120 (13.3)	
**Information seeking role (n=1150), n (%)**				<.001
	Active role	697 (60.6)	240 (20.9)	
	Passive role	72 (6.3)	141 (12.3)	
**Decision making role (n=1147), n (%)**				.003
	Active role	700 (61.0)	342 (29.8)	
	Passive role	55 (4.8)	50 (4.4)	
**Total unmet needs (n=1203), mean (SD)**		4.62 (6.97)	3.58 (6.12)	.011

### Multivariable Logistic Regression

All factors found to be significantly associated with health-related internet use in the univariable analyses were entered into the model. Table 4 shows the results of the multivariable logistic regression analysis. Our results indicate that the odds of using the internet as a health resource were higher among respondents who were active information seekers (OR: 4.57, 95% CI 2.5-8.3), were confident using information from the internet to make health decisions (OR: 3.56, 95% CI 2.27-5.59), had broadband internet access (OR: 1.76, 95% CI 1.14-2.7), and had more unmet supportive care needs (OR: 1.05, 95%CI 1.02-1.09).

**Table 4:** Main effects model resulting from multivariable logistic regression.

Variables	Parameter estimate	Standard error	*P* value	Odds ratio	95% CI for odds ratio	
					Lower	Upper
Active information seekers	1.51	0.28	0.000	4.51	2.60	7.83
Confidence in using internet information to help make health decisions	1.29	0.23	0.000	3.63	2.32	5.67
Broadband internet access	0.58	0.22	0.008	1.78	1.16	2.74
Total number of unmet supportive care needs	0.05	0.02	0.004	1.05	1.02	1.09
Constant	–0.86	0.314	0.006	0.425		

### Most Commonly Used Websites

A total of 22% of respondents (n=308) provided 359 valid responses to the open-ended question “Are there any specific Internet site(s) that you like to go to for information related to prostate cancer or its treatments? If Yes, please specify.” Responses that were not valid consisted of unspecified sites (eg, “health website”, search engines etc). As shown in Table 5, of the 10 categories of websites that were identified, the most commonly reported websites were national cancer agencies, government, or hospitals. The top three most commonly mentioned national cancer agencies or government websites were the Canadian Cancer Society (n=40), Prostate Cancer Canada (n=23), and the American Cancer Society (n=10). The top three most commonly reported hospital affiliated websites were Mayo Clinic (n=51) BC Cancer Agency (n=14) and Johns Hopkins (n=8). American hospital-affiliated websites accounted for over 50% of all responses in this latter category.

**Table 5:** Types of websites most commonly used as a source of prostate cancer information.

Order	Website type	Count, n (%)
1	National cancer agencies and government websites	133 (37.0)
2	Hospital affiliated websites	117 (32.5)
3	For-profit health information websites and news sites	39 (10.9)
4	Websites with user-generated content (eg, Youtube, Wikipedia)	15 (4.2)
5	Online support groups	13 (3.6)
6	Professional medical association websites	12 (3.3)
7	Alternative and complementary therapies	12 (3.3)
8	Academic journals, bibliographic databases	10 (2.8)
9	Personal websites	5 (1.4)
10	Electronic health record websites	3 (0.8)

### Features and Information Wanted in a Website

Respondents were also asked to indicate what features and information they would want in a website for men with prostate cancer and their families. As shown in Table 6, the top three features that over 50% of respondents wanted in such a website were a library of topics (n=893, 65.6%), tools to help select treatment options (n=815, 59.8%), and tools to help navigate the prostate cancer journey (n=698, 51.2%). As shown in Table 7, the information that ≥50% of respondents wanted in such a website included information on prostate cancer treatments (n=916, 65%), what is prostate cancer and its natural progression (n=904, 64%), how to manage side effects (n=858, 61%), personally relevant information (n=769, 55%), and latest research (n=746, 53%).

**Table 6:** Features wanted in a website for men with prostate cancer and their families.

Order	Feature	Count, n (%)
1	A library of topics	893 (65.6)
2	Tools to help me select treatment options	815 (59.8)
3	Tools to help me navigate the prostate cancer journey	698 (51.2)
4	Links to trusted websites	649 (47.7)
5	Tools to help me assess my health status (eg, symptom assessment)	612 (44.9)
6	Tools to monitor/track changes in my health	546 (40.1)
7	Tools to record my health information (eg, PSA^a^)	490 (36.0)
8	Appointment reminders (eg, by email or text)	408 (30.0)
9	Online forum to exchange info and support with other patients	389 (28.6)
10	Show your future care plan (eg, survivorship care plan)	362 (26.6)
11	Access to peer support groups	321 (23.6)
12	Tools to manage appointments (eg, calendar)	306 (22.5)
13	Tools to manage contacts	256 (18.8)

^a^PSA: prostate-specific antigen.

**Table 7:** Information wanted in a website for men with prostate cancer and their families.

Order	Feature	Count, n (%)
1	What prostate cancer treatments are available	916 (67.3)
2	What is prostate cancer and its natural progression	904 (66.4)
3	How to manage side effects	858 (63.0)
4	Information recommended based on my personal situation	769 (56.5)
5	Latest research	746 (54.8)
6	My chances of survival and/or cure	697 (51.2)
7	Explanations of what my doctors told me	627 (46.0)
8	How to obtain a second opinion	623 (45.7)
9	Alternative and complementary therapies	580 (42.6)
10	Wellness programs (eg, exercise, nutrition)	578 (42.4)
11	Information and access to clinical trials	523 (38.4)
12	Information to help my family deal with the prostate cancer	468 (34.4)
13	Emotional support for dealing with prostate cancer	407 (29.9)
14	Community support services in my area	361 (26.5)

## Discussion

### Principal Findings

This study examined the patterns of, and factors associated with, the use of the internet as a source of health information among Canadian men with prostate cancer, and the features and information wanted in a website. A total of 82% of respondents in this sample were internet users. The prevalence of internet use in this sample is consistent with the 2016 internet use rates among Canadians within this age group, which showed that 81% of people aged 65-74 were internet users [12]. Although the majority of the respondents in our sample used the internet, less than half (47%) accessed the internet through broadband and less than 30% accessed the internet with a mobile device, with the most using desktop computers to access the internet. These access patterns are also consistent with current broadband and mobile phone usage rates among Canadian seniors, which are still considerably lower than those in younger age groups [12,25].

Our findings suggest that sociodemographic factors, namely age, education, and income, may not play a significant role in determining health-related internet use among prostate cancer survivors in Canada when other factors are considered. Similarly, a previous analysis [15] of the US National Health Information Trends Survey from 2003 to 2008 showed no statistically significant differences in age, education, or racial/ethnic aspects in health-related internet use patterns among cancer survivors, with the exception of emailing providers [15]. Survivors who were younger and had higher education were more likely to email their providers. Likewise, another analysis [11] of the 2000 Canadian National household internet survey found no association between health-related internet use and age, education, or income. Other studies suggest that there is still a gap in health-related internet use based on age and socioeconomic factors in North America [10,17]. However, these studies focused their analyses on internet users [10,17]. Chou et al [15] reported an independent association with educational attainment in their sample of cancer survivors. Similarly, our univariable analysis showed that age, education, and income were associated with health-related internet use among our sample of prostate cancer survivors.

However, our findings suggest that there is a gap in health-related internet use among prostate cancer survivors in Canada based on broadband access, which may reflect rurality [26]. Previous research has shown that urban Canadians are 1.5 times more likely to use the internet than rural Canadians [26]. In our model, respondents who had broadband access were 2.74 times more likely to use the internet as a health resource than those who did not. Broadband, as opposed to traditional dial-up, provides improved internet services, including faster browsing and downloading as well as telephone, radio, and videoconferencing. Not having access to broadband considerably limits one’s quality of internet and access to essential services. In 2000, Johnson et al [11] did not find an association between high-speed internet and health-related internet use in the Canadian national household internet survey. However, longer duration of internet use was associated with decreased likelihood of using the internet as a health resource [11]. Residential broadband was launched in Canada in 1998; hence, most Canadians would have been using dial up then, which would have been costly and unstable over longer periods of use [27]. In a 2015 qualitative study, patients with prostate cancer living in remote areas of British Columbia explained that they did not like to use the internet as a health resource because it was not reliable [28]. In 2016, Canada’s telecom agency declared broadband a basic service [29], along with a commitment to increase targets for download and upload speeds and extend access to regions without access. Our findings provide further evidence in favor of universal broadband internet access. Future research should investigate the impact of this policy.

In our model, the factor associated with the highest odds of using the internet as a health resource was being an active information seeker. Respondents who were information seekers were 4.6 times more likely to use the internet as a health resource than passive information seekers. A 2018 systematic review [30] of men’s health-seeking behavior that largely focused on prostate cancer found that the internet was the primary source of information for active information seekers. We assessed active information seeking by asking respondents to indicate who looked for information about prostate cancer and its treatment for them. The options included (1) “I did not want any information,” (2) “someone else did most or all of the looking,” (3) “I did some of the looking and someone else did some of it for me,” and (4) “I did most or all of the looking myself.” We dichotomized the responses to create a binary variable by combining (1) and (2) to reflect passive information seekers and (3) and (4) to reflect active information seekers. This variable reflects the difference between two coping styles categorized by Miller as “Monitors” and “Blunters” [31]. Monitors focus on acquiring information to help them problem solve and reduce uncertainty and are less satisfied with the information they receive from health care providers [31]. Blunters use distraction to avoid threatening information and are typically satisfied with the amount of information they receive from their health care providers [31]. Research suggests that psychoeducational interventions for cancer patients are most effective when the level and type of information are consistent with the individual's monitoring style and the demands of the health threat [32].

The factor next most strongly correlated with health-related internet use was eHealth confidence. Respondents who were confident using information from the internet to help make health decisions related to their prostate cancer were used 3.6 times more likely to use the internet as a health resource. eHealth confidence was measured using the final item of the eHEALS eHealth literacy measure [20]. eHealth literacy is defined as the ability to seek, find, understand, and appraise health information from electronic sources and apply that knowledge to solve a health problem or make a health-related decision [20]. The final item of the eHEALs scale is intended to capture one’s confidence in using information from the internet to help make health decisions. The level of agreement with this item in this sample (40.2%) is similar to that reported in a sample of older adult internet users in the United States (43.1%) [16]. Other studies have found an association between eHealth literacy, confidence, and health-related internet use. In 2003, Mead et al [33] found that positive outcome expectancy (eg, patients’ belief that it would enable them to better deal with their health), previous use of health websites, and positive self-efficacy (patients’ confidence in their ability to use the technology) were the strongest predictors of patient-reported interest in getting health information from the internet in the United Kingdom [33]. Ten years later, Tennant et al [16] reported that greater eHealth literacy was associated with greater use of Web 2.0 and social media for health information among a sample of Americans.

As shown in other studies of cancer patients’ use of online resources [34], unmet supportive care needs were also associated with health-related internet use. For each unit increase in unmet supportive care needs, the odds ratio of using the internet as a health resource increased by a factor of 1.05. This may suggest that use of the internet by patients with prostate cancer is problem focused, driven by a need to find information to address a specific issue or problem. This finding has important implications for the design and evaluation of health information websites, as it suggests that websites should be designed to address the specific supportive care needs of patients. Respondents in our sample indicated that they want prostate cancer websites to include information on treatment options and side effects and how to manage them, as well as features that help them make treatment decisions and navigate the prostate cancer journey. This type of task-oriented internet use that is motivated by specific needs also suggests that once prostate cancer patients’ needs are met by a Web resource, they may be unlikely to keep using it, unless a new need arises. Therefore, discontinued use of a Web resource may not necessarily be a failing of the site’s design but rather a potential logical reaction to changing needs and circumstances [35]. Hence, website evaluation metrics should focus on assessing whether users’ specific needs have been met.

Problematically, our findings revealed that the majority of prostate cancer patients’ in our sample lacked confidence in using information from the internet to make health decisions related to their prostate cancer. Research suggests that seniors have lower eHealth literacy than their younger counterparts [36]. One study found that seniors felt confident in their ability to use the internet to search for health information, but less confident in their ability to assess the quality of that information [16]. eHealth literacy interventions may help. An intervention study by Xie [37] showed that a 2-week eHealth literacy training program increased seniors knowledge, skills, and eHealth literacy efficacy [37]. At the same time, there is a need to improve the quality of prostate cancer websites. When systematically reviewed by Black and Penson in 2006 [38] and again by Kobes et al in 2018 [39], websites containing information on prostate cancer were found to be lacking in currency, attribution, balance of evidence, and comprehensiveness. In another study, only 3 of 62 websites containing information on prostate cancer treatment options were written below the recommended high school reading level [40]. In yet another study, Genova and Bender [41] found that websites containing information on prostate cancer treatment did not present information in a useful or credible way for patients. Of the 35 websites examined, the average communication quality score was 24 of 50, and less than 50% included content on risk communication, usefulness, and scientific value.

This study has certain limitations. First, as this study was cross-sectional, only association and not causation can be inferred. We aimed to obtain a representative sample of prostate cancer survivors in Canada by recruiting a random sample of patients with prostate cancer from four provincial cancer registries. It is possible that the individuals who chose not to participate in the survey had different characteristics, experiences, and attitudes regarding using the internet as a health resource. It is also possible that patterns of internet use among prostate cancer survivors have increased since then. Nonetheless, our findings are comparable to other studies involving patients with prostate cancer and current Canadian population norms. Our study was also limited by the use of the opt-in methodology used by one provincial cancer registry, which reduced the overall response rate. We encourage researchers to argue in favor of opt-out recruitment methods for future research. We also assessed only one parameter of the validity of the eHealth confidence measure. Further work is needed to establish the validity of this single item as a measure of eHealth confidence. Lastly, we did not ask for respondents’ views on patient portals or personal health records, which are growing in demand. Future research should also explore the internet use trends among French-speaking Canadian patients with prostate cancer and those from diverse ethnic backgrounds.

### Conclusion and Implications

The internet has the potential to serve as a health information resource for the majority of Canadian men with prostate cancer. In our sample, over two-thirds of patients with prostate cancer used the internet as a health resource. However, one-third of patients with prostate cancer in our sample did not want to use the internet as a health resource, and more than half were not confident in using the internet to make health decisions. Clinicians and educators should not assume that because of their age, education, or income, men with prostate cancer are not interested in using the internet as a health resource. Rather, they should be aware of the importance of being an active information seeker, having confidence in using information from the internet to make health decisions, and having broadband access and unmet supportive care needs as determinants of health-related internet use. These findings also show that men are looking for information on the internet about treatment options, disease progression, and management of side effects, and want websites to include features that help them make treatment decisions and navigate the prostate cancer journey. Future work should examine eHealth literacy interventions as a means to boost men’s confidence in using information from the internet for prostate cancer decision making and design websites that include the information and features that patients with prostate cancer want most.

## Abbreviations

eHEALS: eHealth Literacy Scale
HINTs: US Health Information National Trends survey
OR: odds ratio
STROBE: Strengthening the Reporting of Observational Studies in Epidemiology
